# Patient descriptions of loss of control and eating episode size interact to influence expert diagnosis of ICD-11 binge-eating disorder

**DOI:** 10.1186/s40337-020-00342-z

**Published:** 2020-11-23

**Authors:** Laura A. Berner, Robyn Sysko, Tahilia J. Rebello, Christina A. Roberto, Kathleen M. Pike

**Affiliations:** 1 Department of Psychiatry, Icahn School of Medicine at Mount Sinai, New York, NY USA; 2 Department of Psychiatry, Columbia University Irving Medical Center, New York, NY USA; 3 Department of Medical Ethics and Health Policy, Perelman School of Medicine, University of Pennsylvania, Philadelphia, PA USA

**Keywords:** Binge eating, Loss-of-control eating, Overeating, Binge-eating disorder, Diagnosis and classification, ICD-11

## Abstract

**Background:**

Although data suggest that the sense of “loss of control” (LOC) is the most salient aspect of binge eating, the definition of LOC varies widely across eating disorder assessments. The WHO ICD-11 diagnostic guidelines for binge eating do not require an objectively large amount of food, which makes accurate LOC diagnosis even more critical. However, it can be especially challenging to assess LOC in the context of elevated weight status and in the absence of compensatory behaviors. This ICD-11 field sub-study examined how descriptions of subjective experience during distressing eating episodes, in combination with different eating episode sizes, influence diagnoses of binge-eating disorder (BED).

**Method:**

Mental health professionals with eating disorder expertise from WHO’s Global Clinical Practice Network (*N* = 192) participated in English, Japanese, and Spanish. Participants were asked to select the correct diagnosis for two randomly assigned case vignettes and to rate the clinical importance and ease of use of each BED diagnostic guideline.

**Results:**

The presence of LOC interacted with episode size to predict whether a correct diagnostic conclusion was reached. If the amount consumed during a typical distressing eating episode was only subjectively large compared to objectively large, clinicians were 23.1 times more likely to miss BED than to correctly diagnose it, and they were 9.7 times more likely to incorrectly diagnose something else than to correctly diagnose BED. In addition, clinicians were 10.8 times more likely to make a false positive diagnosis of BED when no LOC was described if the episode was objectively large. Descriptions of LOC that were reliably associated with correct diagnoses across episodes sizes included two that are similar to those already included in proposed ICD-11 guidelines and a third that is not. This third description of LOC focuses on giving up attempts to control eating because perceived overeating feels inevitable.

**Conclusions:**

Results highlight the importance of detailed clarification of the LOC construct in future guidelines. Explicitly distinguishing LOC from distressing and mindless overeating could help promote consistent and accurate diagnosis of BED versus another or no eating disorder.

**Supplementary Information:**

**Supplementary information** accompanies this paper at 10.1186/s40337-020-00342-z.

## Plain English summary

A sense of “loss of control” (LOC) is the feature that distinguishes binge eating from other kinds of overeating. LOC definitions vary widely, and this study aimed to examine how different descriptions of LOC influence whether a diagnosis of binge-eating disorder (BED) will be assigned. An internet-based vignette study was conducted through the World Health Organization’s Global Clinical Practice Network; 192 mental health professionals with self-reported expertise in eating disorders participated. The size of the eating episode interacted with the presence of LOC to affect whether a correct diagnosis was given to the hypothetical patients. For episodes in which the amount eaten was within normal limits (i.e., only subjectively large) and LOC was described, clinicians were 23.1 times more likely to miss BED and 9.7 times more likely to incorrectly assign another diagnosis than to correctly diagnose BED. If the amount eaten was objectively large but there was no LOC, clinicians were 10.8 times more likely to make a false positive diagnosis of BED. Thus, how LOC is described is important to promote consistent and accurate diagnosis of BED versus another or no eating disorder.

## Introduction

The behavioral disturbance of binge eating has been defined by two essential dimensions in the *Diagnostic and Statistical Manual of Mental Disorders* (DSM) and International Classification of Diseases (ICD). Beginning with the *DSM-III*, the presence of binge eating required the consumption of an objectively large amount of food (e.g., unusually large for the circumstances) coupled with the experience of loss of control (LOC). However, in clinical practice, descriptions of the amount of food eaten during binge eating episodes (i.e., episode size) is highly variable [1]. Individuals who report subjective binge eating, or consuming an amount of food that is within normal limits but is perceived as large while experiencing LOC, describe comparable levels of distress and indicators of psychopathology as individuals who report objective binge eating [2–5]. Thus, in the 11th Revision of the ICD (ICD-11), the guidelines for defining a binge episode were updated such that the critical characteristic of binge eating is a sense of LOC [6]. The guidelines specifically note that “binge eating episodes may be ‘objective,’ in which the individual eats an amount of food that is larger than most people would eat under similar circumstances, or ‘subjective,’ which may involve eating amounts of food that might be objectively considered to be within normal limits but are subjectively experienced as large by the individual.” [6]. These differ from *DSM-5* criteria for binge eating, which still require both the consumption of an objectively large amount of food and a sense of LOC [7] (see Table S1).

Regardless of the amount of food consumed, the sense of LOC during eating episodes predicts significant distress, impairment, and clinical outcome, including the development of eating disorders, weight gain, and less weight loss when LOC persists or develops after bariatric surgery [8, 9]. Measures have been developed to dimensionally assess LOC severity [10, 11]; however their language and constructs vary, and no clinical “cutoff scores” are provided. As such, there is no standard method to diagnose LOC. Accurate assessment of LOC is particularly critical because, as aforementioned, the recently adopted ICD-11 guidelines do not require an objectively large amount of food in the diagnosis of binge eating.

Although supported by research and clinical practice, the omission of a large episode size guideline could increase the risk of binge eating misdiagnosis. One specific concern is that clinicians unfamiliar with updates in ICD-11 may underdiagnose binge eating in individuals who experience LOC during eating episodes that are not objectively large. This is problematic because LOC strongly contributes to more negative psychosocial, behavioral, and weight outcomes. Individuals with high LOC who could benefit significantly from receiving appropriate care may not be referred for treatment. In an initial field study, even clinicians with expertise in eating disorders from around the globe were significantly less accurate in diagnosing ICD-11 bulimia nervosa (BN) when subjectively large LOC episodes rather than objectively large LOC episodes were described [12]. As binge-eating disorder (BED) excludes the compensatory behaviors seen in BN that may more obviously signal an eating disorder, and it does not require the presence of any other maladaptive eating behaviors besides regular LOC eating in order to be diagnosed, clarifying the definition of LOC is particularly important for the prevention of BED underdiagnosis.

A second major concern is that other kinds of distressing eating could be conflated with LOC eating, leading to binge eating overdiagnosis. For example, among adults with overweight or obesity, several maladaptive eating behaviors that are not characterized by LOC are commonly reported, including grazing, chaotic or disorganized eating, stress-related or emotional eating, eating much more rapidly than normal, and mindless eating [13, 14]. The ICD-11 guidelines note that individuals with obesity who report overeating patterns that do not meet the definition of binge eating should not be diagnosed BED. However, it may be difficult to distinguish individuals who are binge eating from those who are only distressed by maladaptive eating behaviors.

Overall, accurate LOC diagnosis has significant implications for BED diagnostic sensitivity and specificity, and additional guidance on the diagnosis of LOC may be helpful for both clinical practice and research studies. The current study aimed to examine the influence of LOC and size of the eating episode on how clinicians assigned a diagnosis of BED, another diagnosis, or no eating disorder, according to ICD-11 guidelines. In addition, because accurate clinical detection of LOC is essential for the diagnosis of all eating disorders characterized by binge eating, we explored which descriptions of LOC most often promoted correct vs. incorrect diagnostic conclusions, across episode sizes. Mental health professionals with eating disorder expertise from the World Health Organization’s (WHO’s) Global Clinical Practice Network completed a survey with two randomized clinical vignettes and associated diagnostic questions. Vignettes were identical for all participants, except for the description of LOC eating, or lack thereof, and whether the episode size was objectively or subjectively large. We hypothesized that LOC and episode size would interact to predict diagnosis. Specifically, we predicted that vignettes that did not include descriptions of LOC would be more likely to be incorrectly associated with a BED diagnosis in the context of an objectively large amount of food, whereas quantity of food consumed would have less influence on BED diagnosis for vignettes that included clear descriptions of LOC.

## Method

### Participants

Participants were recruited globally from a pool of health professionals (the “Global Clinical Practice Network,” or GCPN, http://gcp.network) who had previously registered and provided detailed demographic and professional information using an online Qualtrics software-based survey [15]. Clinicians in the GCPN who endorsed expertise in Feeding or Eating Disorders and indicated that they were an advanced speaker in English, Spanish, or Japanese (*n* = 644) were invited to participate in this sub-study. Of those eligible, 208 (32.3%) responded to the survey link and began the study.

### Procedure

Participants were first presented with and asked to review an abbreviated version of proposed ICD-11 clinical diagnostic guidelines for BED and BN as of January 2018: For both disorders, definitions of LOC and any phrases related to episode size were omitted from the provided guidelines (see Table S1 for a summary of the language removed from the proposed guidelines in the version that was presented to raters). As such, the essential (required) feature of “Frequent, recurrent episodes of binge eating (e.g., once a week or more over a period of three months)” was described only as follows: “Binge eating is defined as a distinct period of time during which the individual experiences a loss of control over his or her eating behaviour. Other characteristics of binge eating episodes may include eating alone because of embarrassment, or eating foods that are not part of the individual’s regular diet.”

Next, in a repeated-measures design, participants were presented with two cases from a pool of 28 vignettes that varied based on the adult presenting for treatment (the “vignette base”; see Supplement for the two vignette bases used in this study), distressing eating episode size (objectively or subjectively large), and experience during distressing eating episodes (seven descriptions of LOC or no LOC; see Table 1). Across the two presented cases, each participant saw the two vignette bases, one paired with a subjectively large episode and one paired with an objectively large episode, and two different experience descriptors. The order of the vignette bases and episode sizes were independently randomized, and the experience descriptors were assigned using random selection without replacement.

**Table 1:** Descriptions of experiences during LOC and non-LOC eating episodes

**Non-LOC Descriptors**	**Based on**
“I’ll be watching TV while I’m eating, so I don’t really taste the food or notice what’s happening, but I just keep going back for more. Before I know it, all the food is gone, and I’ve eaten more than I planned.”	• Chen & Safer, 2010 [16] • Mindful Eating Questionnaire [17]
“I’ve never tried to stop myself; I like the taste of it, so I just keep eating.”	Clinical descriptions from adults seeking weight loss treatment
**LOC Descriptors**	**Based on**
“During times like those, I feel helpless to control my urges to eat.”	Binge Eating Scale [18]
“I feel this drive to keep eating once I get started.”	Eating Disorder Examination [19]
“It’s hard for me to stop eating when I eat like that.”	• Eating Disorder Inventory-3 [20] • Three-Factor Eating Questionnaire [21] • Eating Disorder Examination [19]
“I feel like I can’t stop or limit the amount of food or the type of food I’m eating.”	• Questionnaire on Eating and Weight Patterns-5 [22] • DSM-5 [7]
“I don’t really try to control my eating anymore. Eating like that is pretty much inevitable.”	Eating Disorder Examination [19]

*LOC* loss of control

The two vignette bases both described adult women with body mass indices (BMI) over 25 kg/m^2^ who self-reported high levels of distress about their weight and episodes of what they called “binge eating.” Both case examples denied compensatory behaviors. The vignettes had been validated by eight independent eating disorder expert raters who confirmed that, if actual LOC was described, the individuals in both vignettes met all ICD-11 guidelines for BED diagnosis.

These raters also confirmed the classification of the two episode size descriptions. The episode characterized as within normal limits (i.e., subjectively large) was described as “5 small caramel candies with 2 standard-size scoops (i.e., approximately 1 cup or 214 g total) of ice cream.” The episode characterized by an objectively large amount of food was described as “6 slices of regular-crust, cheese pizza and 2 large orders of French fries.”

Five LOC descriptors were created based on items from well-validated measures of binge eating (see Table 1). In addition, we included two descriptions of distressing eating experiences that are not LOC. One specifically focused on mindless eating. As described by Chen & Safer [16], mindless eating is “not attending to one’s eating (e.g., eating popcorn while watching TV and finding that one has finished the bowl without being aware of this occurring). Unlike binge eating, a loss of control is not experienced in mindless eating” (p. 303). Based on this description, and the items of the Mindful Eating Questionnaire [17], we developed a description of mindless eating without LOC. Finally, we included a description of no past attempt to control eating behavior. This was informed by descriptions of overeating from the co-authors’ clinical experiences treating and assessing adults seeking weight loss treatment (Table 1).

For each vignette, participants were asked to select a diagnosis from a list of BN, BED, another feeding or eating disorder, no diagnosis, or a different diagnosis not on the list (in which case they could specify which diagnosis they selected in a text box). Participants were able to review the diagnostic guidelines and vignette while making their selection. After diagnostic selection, participants were asked to indicate whether each essential feature of their chosen diagnosis was present in the specific case vignette. After reviewing the essential features, participants had the option to change their response and select a different final diagnosis. Participants then reviewed a second clinical vignette and repeated the process. After viewing both vignettes, participants were asked to rate the clinical importance and ease of use of each of the five main BED diagnostic guidelines on a scale of 1–4 (1 = not at all, 4 = extremely) and provide additional information about how often they encounter individuals with BED through direct clinical contact.

### Statistical analysis

Given the non-independence of our categorical data (each clinician made diagnostic decisions about two vignettes), we tested our hypotheses about the prediction of a correct or incorect diagnosis using binomial logit link generalized estimating equations (GEE) with subject as a repeating factor in R. An autoregressive correlation structure best fit the data ranked by QIC [23]. Multinomial GEEs examined the prediction of specific diagnostic conclusions. Vignette base was unrelated to whether a diagnosis was correct or whether the final diagnosis was BED (*p*s > 0.05). However, BED was more likely to be diagnosed on the first vignette than the second (*B* = 0.84, *SE* = 0.25, *p* = 0.0007), and specifically, BED was more likely to be correctly diagnosed than missed on the first vignette than the second (*B* = 0.99, *SE* = 0.37, *p* = 0.008). Therefore, all subsequent models included vignette number as a covariate.

We ran the following models to test our main hypotheses: (1) an overall model testing whether size (subjectively large, objectively large) and experience descriptor (LOC, no LOC) interact to predict whether a correct diagnosis is made, (2) a model testing whether size (subjectively large, objectively large) predicts specific diagnostic decisions when LOC is present (correct diagnosis of BED [reference category], missed BED diagnosis, or incorrect other diagnosis) and (3) a model testing whether size (subjectively large, objectively large) predicts specific diagnostic decisions when LOC is absent (correct conclusion of no diagnosis [reference category], false positive BED diagnosis, false positive other diagnosis). A fourth model examined whether patients’ descriptions of their subjective experience (7 possibilities) and size (subjectively large, objectively large) interact to predict whether a correct diagnosis is made. Alpha was set at 0.0125 to Bonferroni correct for four tests. Finally, exploratory repeated-measures analyses of variance compared ratings of importance and ease of use of each of the BED guidelines.

## Results

Of the 208 clinicians who began the survey, 192 completed questions about at least one vignette and were included in analyses. A total of 188 completed questions about two vignettes. Participant characteristics are shown in Table 2. Most were physicians or psychologists and were from Europe or North America. Participants mostly identified as female (66.1%) with a mean age of 49.2 (*SD* = 10.8) and roughly 18 years of clinical experience. Of note, the modal frequency of encountering patients with subthreshold or threshold BED was less than once per month (34.4% of the sample).

**Table 2:** Participant Characteristics (*N* = 192)

**WHO Global Region**	***N*** **(%)**
Africa	2 (1.0)
USA and Canada	47 (24.5)
Latin America/Caribbean	16 (8.3)
Eastern Mediterranean	5 (2.6)
Europe	93 (48.4)
Southeast Asia	1 (0.5)
Western Pacific—Asia	20 (10.4)
Western Pacific—Oceania	8 (4.2)
**Demographics**	***N*** **(%) or Mean (SD)**
Female:Male	127:65 (66.1:33.9)
Age	49.2(10.8)
**Clinical Profession**	***N*** **(%)**
Medicine	72 (37.5)
Psychology	99 (51.6)
Nursing	3 (1.6)
Social Work	7 (3.6)
Other	4 (2.1)
Counseling	6 (3.1)
Occupational Therapy	1 (0.5)
**Clinical Experience**	**Mean (SD)**
Years of Experience	18.0 (10.4)
Frequency of encountering individuals with subthreshold or threshold BED (scale of 1–5, 1 = never, 5 = very frequently, multiple times per week)^a^	3.0 (1.1)

*WHO* World Health Organization; ^a^*n* = 187

### Does the influence of LOC presence or absence on correct diagnosis depend on episode size?

LOC presence interacted with episode size to predict whether a correct diagnostic conclusion was reached (*p* < 0.0001; Table 3). When LOC was present, correct diagnoses were most often reached in the context of large episodes (i.e., BED was correctly diagnosed when episodes were large). The most frequent incorrect diagnoses were made when the episode was subjectively large and LOC was present (i.e., BED was missed or incorrectly diagnosed as something else).

**Table 3:** LOC presence and size interact to predict correct diagnosis

Parameter	B	SE	Wald
Intercept	−0.47	0.31	2.26
Vignette Number	−0.77**	0.27	7.86
LOC Presence	1.00**	0.37	7.45
Episode Size	−1.76**	0.58	9.09
LOC Presence x Episode Size	4.34***	0.70	38.23

**p < 0.01, ***p < 0.0001; LOC = loss of control

### Does episode size predict specific diagnostic decisions when LOC is present?

When one of the five true LOC descriptors was presented (see Table 1), episode size predicted specific diagnostic decisions (overall model *p* < 0.0001; Table 4). If the episode was subjectively large compared to objectively large, clinicians were 23.1 times more likely to miss BED than to correctly diagnose it, and they were 9.7 times more likely to incorrectly diagnose something else than to correctly diagnose BED (Table 4).

### Does episode size predict specific diagnostic decisions when LOC is absent?

When one of the two non-LOC descriptors was used, episode size also predicted specific diagnostic decisions (overall model *p* = 0.0001). Specifically, clinicians were 10.8 times more likely to make a false positive diagnosis of BED than no diagnosis if the episode was objectively large compared to subjectively large (Table 4).

**Table 4:** Episode size is linked to specific diagnostic decisions when LOC is present and absent

Parameter	B	SE	B	SE
**LOC is Present**	**Logit 1 (Incorrect Other Dx vs. Correct BED)**		**Logit 2 (BED Miss vs. Correct BED)**	
Intercept	−1.62***	0.31	−1.35***	0.30
Vignette Number	1.04**	0.38	1.52***	0.43
Episode Size	−2.27***	0.45	−3.14***	0.60
**LOC is Absent**	**Logit 1 (False Positive Other Dx vs. Correct No Dx)**		**Logit 2 (False Positive BED vs. Correct No Dx)**	
Intercept	−0.66	0.57	0.84*	0.39
Vignette Number	0.59	0.71	−1.15	0.66
Episode Size	0.40	0.77	2.38**	0.77

*p < 0.05, **p < 0.01, ***p < 0.0001; *BED* binge-eating disorder, *LOC* loss of control, *Dx* diagnosis; reference categories were correct BED diagnosis and a subjectively large episode

### Does the influence of LOC description (or lack thereof) on correct diagnosis depend on episode size?

**Fig. 1:**
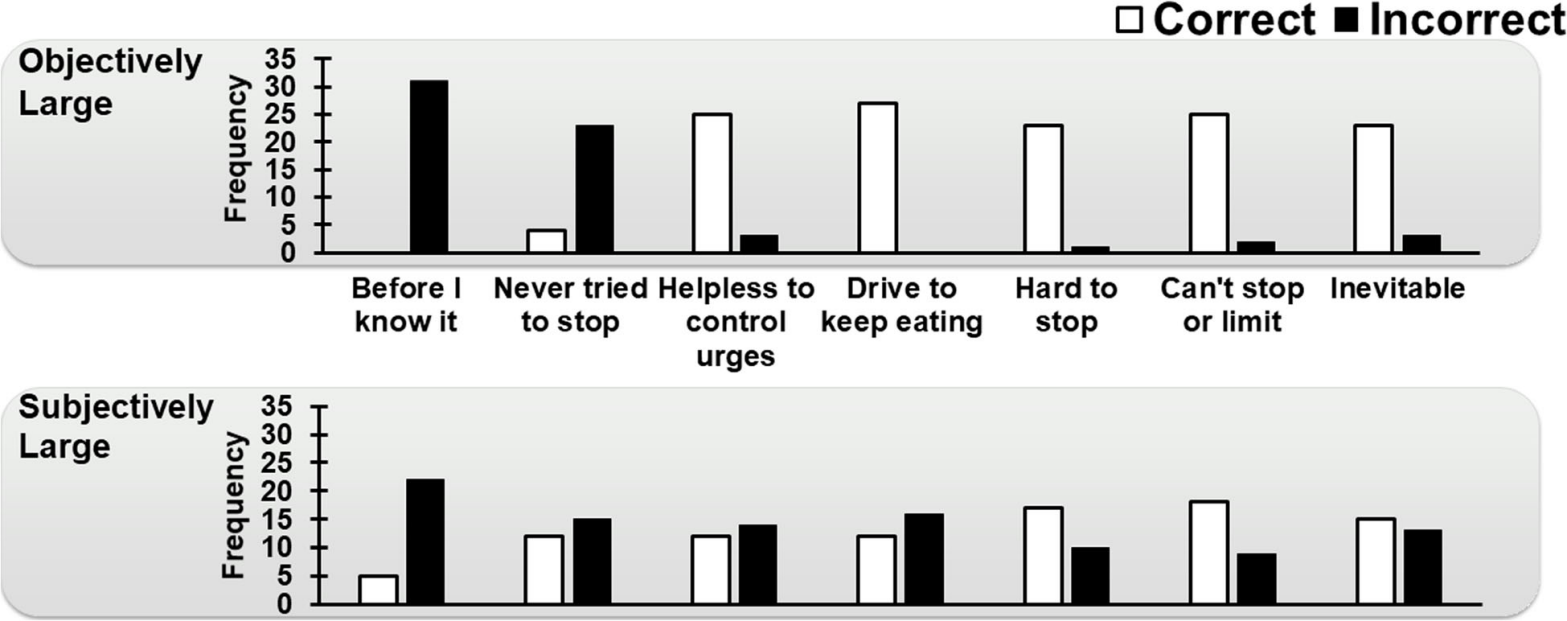
The description of the patient’s experience during the eating episode interacts with episode size to predict diagnostic accuracy (*p* = 0.012). See Table 1 for full descriptions

The description of the patient’s experience during the eating episode interacted with episode size to predict whether a correct diagnostic conclusion was reached (*p* = 0.014; Table 5). As shown in Fig. 1, the only “true LOC” descriptors that were consistently associated with more correct than incorrect diagnostic conclusions across episode sizes were “It’s hard for me to stop eating when I eat like that,” “I feel like I can’t stop or limit the amount of food or the type of food I’m eating,” and “I don’t really try to control my eating anymore. Eating like that is pretty much inevitable.” In contrast, across both objectively large and subjectively large episode sizes, experts almost always made incorrect diagnoses when mindless eating was described, and they made more incorrect than correct diagnoses when no attempt to control eating was described.

**Table 5:** Specific LOC description (or lack thereof) and size interact to predict correct diagnosis

	B	SE	Wald
Intercept	−0.21*	0.81	0.06
Vignette Number	−0.60**	0.22	7.09
Descriptor	0.26***	0.08	11.85
Episode Size	−0.02	0.47	0.01
Descriptor x Episode Size	0.30*	0.12	5.99

*p < 0.05, **p < 0.01; ***p < 0.001; *LOC* loss of control

### Exploratory analyses: how important and easy to use is each diagnostic guideline for BED?

A total of 187 experts provided ratings on the importance and ease of use of the five ICD-11 BED diagnostic guidelines. Importance (*F* (3.56, 186) = 15.04, *p* < 0.0001) and ease ratings (*F*(3.68, 185) = 17.47, *p* < 0.001) differed across guidelines. Post-hoc pairwise comparisons indicated that importance ratings were significantly higher for the sense of LOC over eating than for episode size, binge episode frequency, and distress (*p*s < 0.005). However, experts rated the sense of LOC over eating guideline as the least easy (i.e., most difficult) to apply in a valid and/or reliable way, and these ease ratings were statistically significantly lower than those for binge episode frequency (*p* < 0.001) and the lack of compensatory behaviors guideline (*p* = 0.005).

## Discussion

The results of this study are important for the future diagnosis of BED. Because the ICD serves as the diagnostic system for all WHO member nations, and the 11th ICD revision is the first to include BED, ICD diagnostic guidelines regarding the behavioral disturbance of binge eating require significant attention. The current proposed ICD-11 guidelines explicitly indicate that binge-eating episodes can be comprised of small, normal, or large quantities of food, leaving LOC as their core defining feature. However, because this is a change from the ICD-10 description of binge eating (in BN guidelines) and is distinct from the *DSM-5* definition of binge eating, focused education will likely be necessary to ensure reliability of diagnosis.

Our findings indicate that experienced clinicians are best at recognizing BED when binge-eating episodes are characterized by both LOC and an objectively large amount of food. Although there is great cultural and contextual variation in the definition of an objectively large amount of food, at the extreme, consensus exists. Our results also suggest that experienced clinicians are likely to miss BED when binge-eating episodes are characterized by LOC and a subjectively large amount of food. As individuals with subjectively large LOC episodes experience levels of distress, associated psychopathology, and impairment comparable to those with objectively large LOC episodes [2–5], it is important that these individuals get diagnosed and referred for treatment. The change in the ICD-11 will allow for appropriate detection and referral; however, our findings highlight that clinician training may be vital to ensure that such individuals receive care.

Given the prioritization of LOC in defining a binge eating episode, establishing standard clinical descriptions of LOC is essential. Prior work provides some examples of detailed LOC descriptions and case examples that could be helpful to include in diagnostic training materials (e.g., [24]). Our results suggest that three descriptions of LOC were most reliably associated with correct diagnoses across episode sizes: “It’s hard for me to stop eating when I eat like that,” “I feel like I can’t stop or limit the amount of food or the type of food I’m eating,” and “I don’t really try to control my eating anymore. Eating like that is pretty much inevitable.” These findings may importantly inform future research studies examining BED and other eating disorders characterized by binge eating. The working definition of LOC proposed by Latner and colleagues [11], *DSM-5* diagnostic features for BN and BED, and the proposed ICD-11 guidelines for BN and BED (Table S1 and S2) include the first two of these three LOC descriptors. In addition, the diagnostic features sections of *DSM-5* note that “abandoned efforts” to control inevitable eating should be considered LOC [7]. Familiarity with these standard definitions of LOC may have increased clinicians’ abilities to correctly diagnose these descriptions. Adding all three of these example descriptions to future drafted ICD guidelines could provide valuable supporting detail for diagnosticians.

In addition to clinical guidance on reliable descriptions of LOC, it will also be essential for clinical training and research to provide guidance on what LOC is not. In particular, it is important to maintain a boundary between binge eating and overeating. Our results suggest that a high percentage of even expert clinicians confuse binge eating with mindless overeating without LOC and overeating without any attempt to stop or LOC. To reduce this confusion, it may be helpful for guidelines to explicitly note that these patterns of overeating do not meet the definition of binge eating.

Of note, previous studies have suggested that subjective binge episodes are clinically meaningful, but have low reliability [25–27]. Improved diagnostic guidelines, assessment tool instructions, and new measures (e.g., the Eating Loss of Control Scale and the Loss of Control over Eating Scale) may help increase reliability and improve diagnosis and severity assessment across disorders characterized by binge eating [10, 11, 28, 29]. Our findings could help inform the refinement of these assessment tools to measure LOC. For example, although eating disorder experts who contributed to the development of the Loss of Control over Eating Scale (LOCES [11];) highly rated items describing mindless eating as “covering or reflecting” LOC, these mindless eating items had lower corrected item-total correlations [11], and one study found that the LOCES-Brief, which excludes these mindless eating items, provided a better fit to data from both clinical and non-clinical samples [28]. The current results more explicitly suggest that conflation of mindless and LOC eating may be a common cause of binge eating misdiagnosis. As such, diagnostic and research tools that clearly distinguish LOC from the mindlessness that it can sometimes co-occur with may be particularly helpful.

### Strengths and limitations

The multilingual, global sample is significant strength of the study. Prior research has asked clinical and expert populations to explicitly define and describe LOC; however, our vignette-based, repeated-measures design mimicked real-world diagnostic decisions clinicians and researchers face when assessing what individuals describe as a “binge.” As such, this first examination of the interacting influences of LOC and episode size on eating disorder diagnosis has implications directly relevant for clinical and research training and practice.

Several study limitations also should be noted. First, both patient vignettes were women, limiting the generalizability of our findings. BED is the most common eating disorder among men [30], and the influences of episode size and LOC descriptions on diagnosis may be even more complicated in men or other genders [31]. Second, as this study was focused on BED, both vignettes explicitly excluded compensatory behaviors. However, larger binge eating episodes may be linked to a stronger relationship between LOC and purging frequency (Forney et al., 2016), suggesting that the influence of LOC description and size on diagnostic conclusions may be more complex with the addition of purging to the clinical picture. Third, clinicians with eating disorder expertise were asked to make decisions in the context of brief vignettes, not a real clinical sample, and explorations of variation by country or world region were not possible with our modest sample size. Results may not generalize to non-expert clinicians or to individual patients or specific local populations or languages. Unexpectedly, participants diagnosed BED more frequently in the first vignette, regardless of the size or episode description included. As such, future research is needed to determine whether order effects exist in clinical or research diagnostic practice (e.g., whether the first patient of the day may be more likely to receive a correct than a missed diagnosis of BED).

## Conclusions

Accurate and reliable assessment of binge eating is crucial for the diagnosis of BED and other eating disorders. The ICD-11 prioritizes the role of LOC over the amount of food consumed in the behavioral disturbance of binge eating. Although this guideline was established based on research and clinical data, there is currently no brief, standardized diagnostic strategy for LOC. Clinical training focused on the changes in the ICD-11 regarding binge eating and clear clinical and research standards for LOC assessment will be critical to increasing diagnostic consensus.

## Supplementary Information

**Additional file 1: Table S1.** ICD-11 Guidelines as of January 2018 and *DSM-5* Criteria for Binge Eating. **Table S2.** Removed Phrases from January 2018 ICD-11 Proposed Guidelines for Binge Eating in the Current Study.

## Data Availability

Requests for the datasets analyzed in the current study should be submitted to the corresponding author.

## Abbreviations

BED: Binge-eating disorder
BN: Bulimia nervosa
DSM-5: Diagnostic and Statistical Manual of Mental Disorders, 5th Edition
ICD: International Classification of Diseases and Related Health Problems
LOC: Loss of control
WHO: World Health Organization
GEE: Generalized estimating equations
