# Supplementary material for: Patient descriptions of loss of control and eating episode size interact to influence expert diagnosis of ICD-11 binge-eating disorder
Source: J Eat Disord. 2020 Nov 23;8:71. doi: 10.1186/s40337-020-00342-z (PMC7682053; doi:10.1186/s40337-020-00342-z)
Supplement: Supplementary file 1 — Additional file 1: Table S1. ICD-11 Guidelines as of January 2018 and DSM-5 Criteria for Binge Eating. Table S2. Removed Phrases from January 2018 ICD-11 Proposed Guidelines for Binge Eating in the Current Study. [file 40337_2020_342_MOESM1_ESM.docx]

**Supplement**

**Table S1. ICD-11 Guidelines as of January 2018 and *DSM-5* Criteria for Binge Eating**

| **ICD-11 Guidelines** | **DSM-5 Criteria** |
| --- | --- |
| Binge eating is defined as a distinct period of time during which the individual experiences a loss of control over his or her eating behaviour. A binge eating episode is present when an individual eats notably more or differently than usual and feels that (s)he is unable to stop eating or limit the type or amount of food eaten.  Binge eating episodes may be ‘objective’, in which the individual eats an amount of food that is larger than most people would eat under similar circumstances, or ‘subjective’, which may involve eating amounts of food that might be objectively considered to be within normal limits but are considered large by the individual. In either case, the core feature of a binge eating episode is the experience of loss of control over eating. | An episode of binge eating is characterized by both of the following:  1. Eating, in a discrete period of time (e.g., within any 2-hour period), an amount of food that is definitely larger than what most people would eat in a similar period of time under similar circumstances.  2. A sense of lack of control over eating during the episode (e.g., a feeling that one cannot stop eating or control what or how much one is eating). |

**Table S2. Removed Phrases from January 2018 ICD-11 Proposed Guidelines for Binge Eating in the Current Study**

| **ICD-11 Proposed Guidelines for Binge Eating Across BN and BED** | **Language Removed from Guidelines Provided to Study Participants** |
| --- | --- |
| *Essential (Required) Features:*   - Frequent, recurrent episodes of binge eating (e.g., once a week or more over a period of three months). Binge eating is defined as a distinct period of time during which the individual experiences a loss of control over his or her eating behaviour. A binge eating episode is present when an individual eats notably more or differently than usual and feels that (s)he is unable to stop eating or limit the type or amount of food eaten. Other characteristics of binge eating episodes may include eating alone because of embarrassment, or eating foods that are not part of the individual’s regular diet. | A binge eating episode is present when an individual eats notably more or differently than usual and feels that (s)he is unable to stop eating or limit the type or amount of food eaten. |
| *Additional Features:*   - Binge eating episodes may be ‘objective’, in which the individual eats an amount of food that is larger than most people would eat under similar circumstances, or ‘subjective’, which may involve eating amounts of food that might be objectively considered to be within normal limits but are considered large by the individual. In either case, the core feature of a binge eating episode is the experience of loss of control over eating. - Binge eating is typically experienced as very distressing and is often accompanied by negative emotions (e.g., guilt or disgust). Negative emotions related to binge eating (e.g., shame) also typically negatively affect the individual’s self-evaluation. | Binge eating episodes may be ‘objective’, in which the individual eats an amount of food that is larger than most people would eat under similar circumstances, or ‘subjective’, which may involve eating amounts of food that might be objectively considered to be within normal limits but are considered large by the individual. In either case, the core feature of a binge eating episode is the experience of loss of control over eating. |

BN = bulimia nervosa; BED = binge eating disorder

**Case Vignette “Bases”**

**Vignette 1**

**AH**

AH is a 28-year old woman and her body mass index is currently 29.4 kg/m2 (height=5’3”/160 cm, weight=166 lbs/75.3 kg). AH has been overweight since age 12. Over the past few months, AH has been very upset about her eating behaviour. She explained that she has been told since she was a child that she “eats too fast” and said that this continues to be an ongoing problem for her. When asked to provide an example of what AH referred to as “binge eating,” she described a "typical," 1-hour episode, including [Episode Size descriptor]. She said during times like that, her habit of “eating too fast” is particularly noticeable. AH said that this kind of episode occurs when she is home alone, and that although these episodes sometimes occur during holidays, they also occur during many other times. She notices that she sometimes eats in this way when she feels tired, bored, lonely, or anxious, but that it has also happened when she feels “everything else is fine.” AH said she has trouble understanding these episodes because she continues to eat, all in one sitting, when she doesn’t feel hungry, “to the point that my stomach hurts and feels like it’s going to burst.” She added, “[LOC/Non-LOC Descriptor]” Three months ago, this kind of eating was occurring about once per week, two months ago, this kind of eating was occurring about twice per week, and that in the last month it occurred 1-3 times per week. Despite being troubled by these episodes and often feeling depressed and “bad about herself” after them, AH said she had never self-induced vomiting, abused laxatives or diuretics, engaged in driven exercise, or misused any other medications to try to compensate for them.

**Vignette 2**

**GT**

GT is a 33-year old woman who lives alone and said she is “tired of struggling with her weight.” She reported that since she was 10 years old, she has been overweight, and that at her last annual physical, her doctor told her for the first time that she is “obese.” GT’s body mass index is currently 37.7 kg/m2 (height=5’8”/173 cm, weight=248 lbs/112.5 kg). She said that she is “distraught” about both her weight and her eating, which makes her feel like “a terrible person.” She is now at her highest adult weight and reported that her weight has remained fairly stable for the last year. GT has tried several diets, including online programs and low-carbohydrate and low-sugar diets, none of which helped her lose more than 10 lbs. GT attributed her lack of success to episodes of what she called “binge eating” that began approximately two years ago. She said that a typical episode like this occurs almost every other night and would include [Episode Size descriptor]. She said that these episodes often occur over a span of 1-2 hours, usually in the evening, and only in her apartment, because she would be “far too embarrassed” for others to see her eating this way. She feels “extremely guilty” for “undoing” her efforts to lose weight. She added, “[LOC/Non-LOC Descriptor].” GT said that she “tries to eat healthier foods” the day after she has episodes like this, but denied any actions designed to compensate for these eating episodes.
